# Screening and assessment for post-acute COVID-19 syndrome (PACS), guidance by personal pilots and support with individual digital trainings within intersectoral care: a study protocol of a randomized controlled trial

**DOI:** 10.1186/s12879-022-07584-z

**Published:** 2022-08-15

**Authors:** Alina Dahmen, Franziska M. Keller, Christina Derksen, Robin Rinn, Petra Becker, Sonia Lippke

**Affiliations:** 1 Klinikum Wolfsburg, Sauerbruchstraße 7, 38440 Wolfsburg, Germany; 2 Jacobs University Bremen gGmbH, Campus Ring 1, 28759 Bremen, Germany; 3 Julius-Maximilians-Universität, Röntgenring 10, 97070 Würzburg, Germany; 4 Dr. Becker Klinikgruppe, 50968 Cologne, Germany

**Keywords:** Long-COVID, Post-COVID, Post-acute COVID-19 syndrome, Low-threshold screening, Interdisciplinary diagnostic, Symptom assessment, Digital therapy offers, Cross-sectoral care, Medical rehabilitation

## Abstract

**Background:**

Because the clinical patterns and symptoms that persist after a COVID-19 infection are diverse, a diagnosis of post-acute COVID-19 syndrome (PACS) is difficult to implement. The current research project therefore aims to evaluate the feasibility and the practicability of a comprehensive, interdisciplinary, and cross-sectoral treatment program consisting of a low-threshold online screening and holistic assessment for PACS. Furthermore, it aims to evaluate digital interventions and the use of so-called personal guides that may help to facilitate the recovery of PACS.

**Methods:**

This German study consists of a low-threshold online screening for PACS where positively screened participants will be supported throughout by personal pilots. The personal pilots are aimed at empowering patients and helping them to navigate through the study and different treatment options. Patients will then be randomly assigned either to an intervention group (IG) or an active control group (ACG). The IG will receive a comprehensive assessment of physiological and psychological functioning to inform future treatment. The ACG does not receive the assessment but both groups will receive a treatment consisting of an individual digital treatment program (digital intervention platform and an intervention via a chatbot). This digital intervention is based on the needs identified during the assessment for participants in the IG. Compared to that, the ACG will receive a more common digital treatment program aiming to reduce PACS symptoms. Importantly, a third comparison group (CompG) will be recruited that does not receive any treatment. A propensity score matching will take place, ensuring comparability between the participants. Primary endpoints of the study are symptom reduction and return to work. Secondary outcomes comprise, for example, social participation and activities in daily life. Furthermore, the feasibility and applicability of the online screening tool, the holistic assessment, digital trainings, and personal pilots will be evaluated.

**Discussion:**

This is one of the first large-scale studies to improve the diagnosis and the care of patients with PACS by means of empowerment. It is to be evaluated whether the methods utilized can be used for the German and international population.

*Trial registration* ClinicalTrials.gov Identifier: NCT05238415; date of registration: February 14, 2022

**Supplementary Information:**

The online version contains supplementary material available at 10.1186/s12879-022-07584-z.

## Background

By July 15th, 2022, almost 30 million people in Germany have contracted COVID-19 [1]. The diagnosis of COVID-19 is made by detecting the causative SARS Cov-2 virus by PCR testing—the symptoms vary among individuals and affect different organ systems with varying severity and expressions [2, 3]. In addition to COVID pneumonia and lung lesions [4], other organ systems are also affected [5]: For example, neurological symptoms of the so-called neuro-COVID are encephalopathy, infection of the central nervous system, ischemic stroke, and peripheral neurological diseases [6]. Cardiological symptoms can be observed that cause heart failure, myocarditis, cardiomyopathies, cardiac arrhythmias, and heart attacks [7, 8]. Ossification may occur in the musculoskeletal system, especially after long-term ventilation [9]. Psychological consequences can include post-traumatic stress disorder (PTSD), anxiety disorders, and depression [10, 11].

### Diagnosis of the post-acute COVID-19 syndrome

A possible complication or long-term consequence of acute COVID-19 is the occurrence of the post-acute COVID-19 syndrome (PACS, which can be used as synonym for long- and post-COVID) [3]. There are conservative estimates that 4 weeks after the onset of acute COVID-19, about 10% of patients still suffer from symptoms that warrant the diagnosis of post-acute COVID-19 syndrome  [12]. Since there are many different symptoms, this complex diagnosis requires reliable and valid diagnostics for comprehensive symptom clarification, and carefully planned, individual treatment afterwards. Due to the interdisciplinary symptomatology, there is currently no uniform care structure in Germany. Hence, affected patients are mainly treated separately and sequentially by general practitioners and specialists which leads to long waiting times. However, there is no holistic control of a thoroughly necessary intersectoral therapy.

**Fig. 1:**
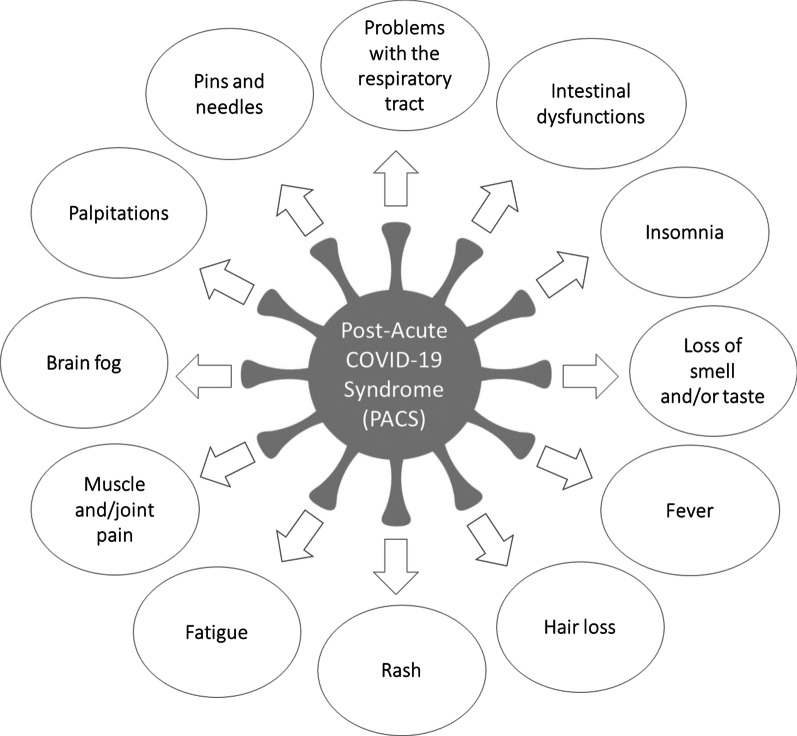
Symptoms of PACS

In contrast to the acute infection, there are currently no validated, uniform diagnostic criteria, so that the development and further refinement of scientific evidence for research into this clinical diagnosis is required [13] and the diagnosis of PACS is made as a clinical case definition, that is, in the presence of symptoms like the ones in Fig. 1 depicted.

Such symptoms that occur after COVID-19 and that cannot be explained otherwise, indicate the diagnosis of a PACS to be probable [14, 15]. There are currently different guidelines regarding the timeline of PACS: According to the guidelines by the UK National Institute for Health and Care Excellence (NICE), symptoms that persist for 4 weeks after the detection of the acute disease or new-onset are referred to as long COVID or subacute/ongoing COVID-19 [16]. If the symptoms persist for more than 12 weeks, they are called post or chronic COVID-19 [16]. According to the definition of the World Health Organization (WHO), PACS can be present if symptoms occur “usually 3 months” after the acute infection and last “at least 2 months”. The S1 guideline “Post-COVID/Long-COVID” of the Association of the Scientific Medical Societies in Germany [17] is based on the following criteria for establishing PACS:

- Symptoms that persist from the acute COVID-19 phase or its treatment,
- symptoms that have led to a new health limitation, or
- new symptoms that have arisen after the end of the acute phase but are understood to be a consequence of COVID-19 disease or worsening of a pre-existing underlying condition.

Among a broad variety of other symptoms, emerging symptoms include fatigue, shortness of breath, cognitive dysfunction, abdominal/chest pain, cough, depression, and altered sense of smell/taste [18]. Many, if not all, of these symptoms, severely affect daily routines in individuals which is why patients desperately seek help from professionals or alternative sources (e.g., [13]). According to a meta-analysis by Augustin et al., PACS is diagnosed when at least one of the following symptoms is present at least 1 to 3 months after the acute infection: inability of smell (anosmia), loss of taste (ageusia), fatigue, or shortness of breath [19]. With these criteria, a PACS diagnosis was made in 27.8% (after 4 months) and 34.8% (after 7 months) of the 958 patients examined [19].

The severity of the course of acute COVID-19 is not a reliable predictor of the occurrence of PACS: An analysis of 28 studies found that 5–36% of outpatients, who had a milder course of COVID-19, and 39–72% of inpatients with a serious trajectory reported post-acute COVID-19 symptoms (PACS) in the first quarter. A high number of symptoms and very explicitly the occurrence of diarrhea or anosmia during the acute infection as well as female sex appear to increase the likelihood for the occurrence of post-acute COVID symptoms [19, 20].

### Consequences for social participation and quality of life

In Bavaria/Germany, where the study is being carried out, about 20,000 patients with PACS are expected according to the data of the Association of Statutory Health Insurance Physicians of Bavaria [21]. Thus, a considerable need for care must be anticipated. In addition to the individual consequences for the affected patients, such as reduced quality of life and poor health, the loss of earnings can have considerable consequences for the individual, their social network, and society in addition to the social security system in Germany (cf. [20]). A survey with 3762 patients found that 7 months after acute COVID-19, only 27.3% were working the same number of hours as before infection, 22.3% were not working at all and 45.6% had reduced their number of hours [18]. Hence, it is crucial that workability and return to work (RTW) are considered in the treatment of PACS. Preventing the loss of employability and restoring workability is a crucial aim in medical rehabilitation which might be an effective treatment option and hence needs to be adapted to the requirements of patients suffering from PACS [22].

Employment and workability are important parts of social participation [23]. Work life poses an imperative indicator of social life which is endangered if societal requirements cannot be fulfilled. Additionally, a reduced cognitive or physical capacity and performance, for example, due to PACS such as fatigue and brain fog can further decrease patients’ social participation, potentially leading to isolation and further deterioration of health and wellbeing. In turn, reduced participation is related to a decrease in mental health and quality of life. Ismael and colleagues have found that approximately 26% of COVID-19 patients suffer from psychological symptoms including stress and PTSD [24] as well as neuro-psychological symptoms such as the inability to concentrate or language impairments [25]. Mental health problems in PACS patients are common and affect patients’ quality of life [26].

A possible management strategy to prevent these detrimental trajectories are health-promoting behaviors including the maintenance of activities of daily living (ADL). It has been hypothesized that self-management in terms of physical activity and healthy diet as part of a behavioral health intervention can positively influence PACS and avoid an exacerbation of symptoms [27]. Hence, it has been added to the WHO self-treatment recommendations [28], but because the exact physiological mechanisms are not sufficiently understood yet, many self-management strategies (such as physical activity or self-medication) should be implemented with caution since some treatments may actually be contraindicative for some patients [29]. Accordingly, patients need to be empowered to consult with their general practitioner and other specialists to get appropriate recommendations and adhere to these recommendations appropriately.

**Fig. 2:**
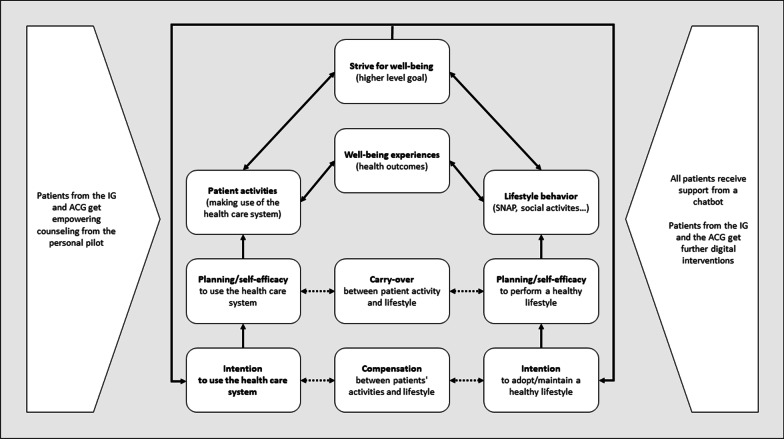
Framework of a successful recovery process building on the Compensatory Carry Over Action Model (CCAM, on basis of Lippke et al. [30])

This will be addressed with a proposed framework of a successful recovery process (see Fig. 2) which builds on the Compensatory Carry Over Action Model (CCAM, [30]). The main idea of the CCAM is that experiences and health outcomes like (physical) activity and (social) participation result from different behaviors such as patient behavior and lifestyle behaviors (e.g., smoking-cessation, nutrition, alcohol abstinence and physical activity abbreviated as SNAP—see Fig. 2).

Patient behavior is the kind of actions patients perform to make use of the health care system (searching for and seeing a doctor, asking questions, providing information, adhering to advice and being able to transfer to other life areas such as lessons learnt regarding symptoms, to prevent further health constraints). These action experiences and resulting health outcomes feed back into behavior management by functioning like outcome expectations. Outcome expectancies are for instance, beliefs and attitudes that our own behavior can improve symptoms instead of only expecting the doctor to fix our health issues. Outcome expectancies are important because they have an impact on higher-level goals. Such higher-level goals drive behavior management centrally as individuals can explicitly or implicitly aim for improving their activity, social participation, and well-being by means of performing actions in that sense (Fig. 2).

### Treatment and management requirements

Due to the wide range of symptoms and limited capacities with specialists, the diagnosis is difficult. Accordingly, a low-threshold screening is of PACS needed to determine PACS, i.e., to support the decision-making processes. Once the diagnosis is made, referrals to different specialists must be made to determine treatment. Since the symptoms of PACS vary greatly among patients [15], the therapy must be adapted to the individual and readjusted in iterations over time, which in turn requires management. For the variety of symptoms, several specialists need to be involved whose respective recommendations must be implemented, hence requiring an effective treatment management approach and patient-centered guidance and care.

In addition to outpatient treatment and therapy, the German guidelines recommend (partial) inpatient medical rehabilitation if there are impairments regarding social participation which require multimodal medical and therapeutic treatment [17]. The aim is buffering long-term health impairments, functional limitations, and endangerment of social participation.

Overall, optimal medical care for patients suffering from PACS with the aim of high treatment quality leads to a considerable effort in the coordination of the treatment process. Successful treatment coordination is currently not guaranteed by the existing structures. That is, in the existing structures of the health care system patients usually, first see their primary care practitioner, who will make the appropriate referral if diagnosis and treatment by a specialist are indicated. Due to the diversity of the symptoms, additional specialized post-COVID outpatient clinics have been set up in many hospitals [31] to which patients also can be referred. Such clinics treat patients partly interdisciplinary and partly specialized in the individual disciplines (cf. [31]). Currently there are 81 of such outpatient clinics listed in Germany (from which 11 are in Bavaria). However, this is not sufficient due to the high rate of patients that are affected from PACS. Furthermore, these outpatient clinics are mainly located in urban areas, so care in sub-urban and rural areas is hardly accessible for patients in need, especially if severely limited with their functionality.

In particular, young affected persons, who previously had not been treated due to (actual or perceived) mild course of the disease, from which it is known to possibly still result in PACS [32], are rarely seen at these clinics [18]. This may lead to a delay in medical support. Accordingly, this could harbor the risk of aggravation and possibly chronification of symptoms, resulting in long-term incapacity to work with limited social participation. This in turn can have a negative impact on the continuation or re-entry into society and lead to economic burdens for the individual and the public in general. Thus, actions are needed. The current project will address these concerns in an exemplary area of Bavaria, Germany with the goal to test and to outline the structure, diagnostic and treatment approach nationwide. The *project aim* is to evaluate a comprehensive, interdisciplinary, and cross-sectoral treatment program consisting of a low-threshold online screening and conducting a holistic assessment for post-acute COVID-19 syndrome (PACS). It also aims to prepare a treatment plan for patients that will be augmented by digital interventions and a so-called personal pilot, who will help patients to navigate through their treatment. In case of the feasibility and effectiveness of the methods applied, our approach will be applied to other regions and countries, too.

### The Research Project

The objective of the present study is the early detection of post-acute COVID-19 symptoms (PACS) and subsequently a long-term and sustainable holistic care and support of patients with PACS to avoid long-term illness and chronification, and to enable social participation.

The long-term goals of the study are the dissemination of validated instruments (i.e.g., the online screening) and newly developed treatment approaches (i.e., the digital interventions and a  personal pilot concept) through communication to central stakeholders such as medical associations, health insurance companies, service providers in the outpatient and inpatient sector as well as professional societies and care institutions. In this way, patient care is to be improved beyond the project duration and care services are to be made more efficient.

Hypotheses, which will be tested with this project, are the following:

1. A short *screening* can detect the presence of PACS.
2. A comprehensive* assessment* can validly and reliably determine rehabilitation or remedial needs and the leading rehabilitation indication and be basis of an individual multidisciplinary treatment plan.
3. The more *individualization* the digital therapy offers, the higher the effectiveness in the treatment of PACS.
4. As contact point for all involved parties, a *personal pilot * empowering the patient suffering from PACS can -by means of an interdisciplinary and intersectoral treatment planning- ensure the sustainable management of interdisciplinary treatment.
5. An *interdisciplinary treatment pathway* and the personal pilots empower the patients, so that the patients receive the appropriate diagnosis and treatment, and to actively participate in their own recovery, ensuring patients recover quickly and sustainably from their PACS conditions, increase their functional capacity, reduce incapacity to work and regain participation.

## Methods

### Study design and setting

**Fig. 3:**
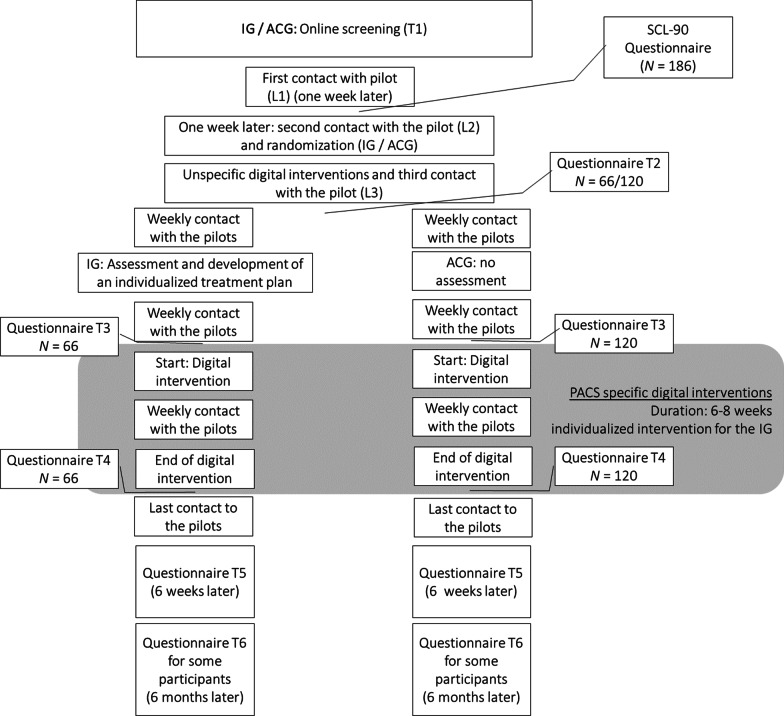
Flowchart of the study design. *IG* Intervention Group, *ACG* Active Control Group

This longitudinal randomized control trial study will take place in Bavaria in Germany and has three study arms. Specifically, patients will be randomly assigned to an intervention group (IG) or an active control group (ACG, see Fig. 3 for the flow chart). Furthermore, a comparison group (CompG) will be recruited. Whereas the CompG will not receive any treatment, the IG and the ACG will have contacts with a *personal pilot* who will help the patients to navigate the study participation as well as through treatment options and help them seek help for further needs. The personal pilot acts as a contact point for patients and service providers and guides patients through the care process. Furthermore, the personal pilots make sure participants will take part in the study questionnaires by continuously reminding the patients in case they did not fill out the questionnaires.

Patients are invited via primary care physicians, support groups, press releases, and social media. Participants may take part when they show signs of PACS symptoms that last longer than 4 weeks after the acute COVID-19 infection. All participants will first take part in a low-threshold *online screening*. Following the online screening, patients receive direct feedback as to whether they are suitable to take part in the study (i.e., whether there are indicators of risk factors of the presence of PACS). Shortly after the positive screening patients from the IG and ACG receive their first contact with a personal pilot.

Patients from the IG and the ACG will then receive unspecific *digital interventions* (e.g., hygiene recommendations etc.). In the IG, this unspecific digital intervention will be made available to bridge the time to an assessment that takes place in a clinic and in which patients will be assessed by a multiprofessional team of health care professionals. After the assessment, patients in the IG receive digital interventions that aim to meet their individual needs in terms of their physical capacity (i.e., physically very resilient, moderately resilient, barely resilient); patients in the ACG receive digital interventions that are individualized based on the most debilitating symptoms (fatigue, neuro-cognitive symptoms, cardio-respiratory). The digital intervention tools consist of training plans on the one hand and additionally, a chatbot will be developed and made available to complement the interventions on the other hand. This chatbot will answer specific questions of participants, for example, about symptoms or who to contact first, and it will also contain short psychological “learn”, “think”, “do”, exercises to improve participants well-being.

In the IG, a 3-day multidisciplinary *assessment* will be carried out promptly at a clinic to determine the individual need for treatment, the results of which are used as the basis for holistic treatment planning. Possible treatment recommendations are the initiation of rehabilitation, the provision of medical aids, specific therapies, the recommendation of digital health applications, and outpatient treatments.

### Blinding

The study design does not provide blinding. Participants, their personal pilots, and health care professionals as well as the researchers who evaluate the data will know about which participants were assigned to which group.

### Data management and anonymity

Data will be collected via unipark.com, an online data collection tool where participants fill out the questionnaires themselves. Within this tool, participants create a unique, pseudonymized study code that they will use at each measurement time point. Furthermore, participants’ full names will be collected within the assessment and during the pilot contacts. The data from the online questionnaires and these surveys will be matched by research associates from the Jacobs University, who will have short-term access to the patient codes and the patients’ true names exclusively for this purpose. The list with the patient codes and the true names will be stored in a protected way and will be destroyed after all matchings have been completed. Furthermore, continuous data backup takes place on protected disks and servers of the institution where the data collection takes place.

### Protocol Version

The current protocol (19. July 2022) is the first version and describes the current status of the procedure.

### Study sample and recruitment

Inclusion and exclusion criteria for the current study are summarized in Table 1.

**Table 1:** Eligibility criteria

Inclusion criteria	Exclusion criteria
Patients perceive PACS symptoms	High need for care as classified according to German health care levels (>/= 2)
Patients who have the necessary prerequisites to participate in the online-screening as well as in telephone or video-conferences with their personal pilots	No mobile device (smartphone, laptop, tablet, or computer) and/or internet connection, no telephone or video conference system, or no sufficient technology literacy to make use of it
Registered in Bavaria, Germany	Occupancy in the healthcare or welfare system or laboratory
Age between 18 and 60 years	PACS treatment or therapy (including rehabilitation)
Willingness to participate in outpatient or (partially) inpatient therapy	Acute COVID-19 infection less than 4 weeks ago
Sufficient German skills	Insufficient literacy of the German language to participate in data collection and digital treatment options Severely limited cognitive, hearing and vision abilities, as linguistic components of the digital offers and auditory stimuli must be understood
Physical conditions that allow participants to take part in the digital intervention exercises	Severely limited physical conditions such as bedriddenness
Patients that do not receive PACS treatment so far	

Patients will be asked to participate in the study after they were positively screened in the initial online screening. The *recruitment* will be carried out in two ways: Firstly, patients are sought to be reached via multiplicators. These include general practitioners (GPs) and specialized primary care physicians, post-COVID outpatient clinics and administrative institutions. GPs and specialists will be informed in advance about the possibility of the screening based on an existing address database. It is assumed that approximately every tenth physician will identify patients as potential candidates and can refer approximately one patient to the study. If 1000 multiplicators are contacted, about 100 potential participants can be screened.

Secondly, patients will be recruited via the use of media, including regular press releases (approx. once a month after more frequent press releases in the beginning of the recruitment), radio podcasts as well as social media posts on Facebook and Twitter. If 20% click on the link to the website of WHO and 20% take part in the screening, 2500 people with PACS are needed to be reached, to recruit 100 study participants.

### Aims, interventions, and innovations

**Fig. 4:**
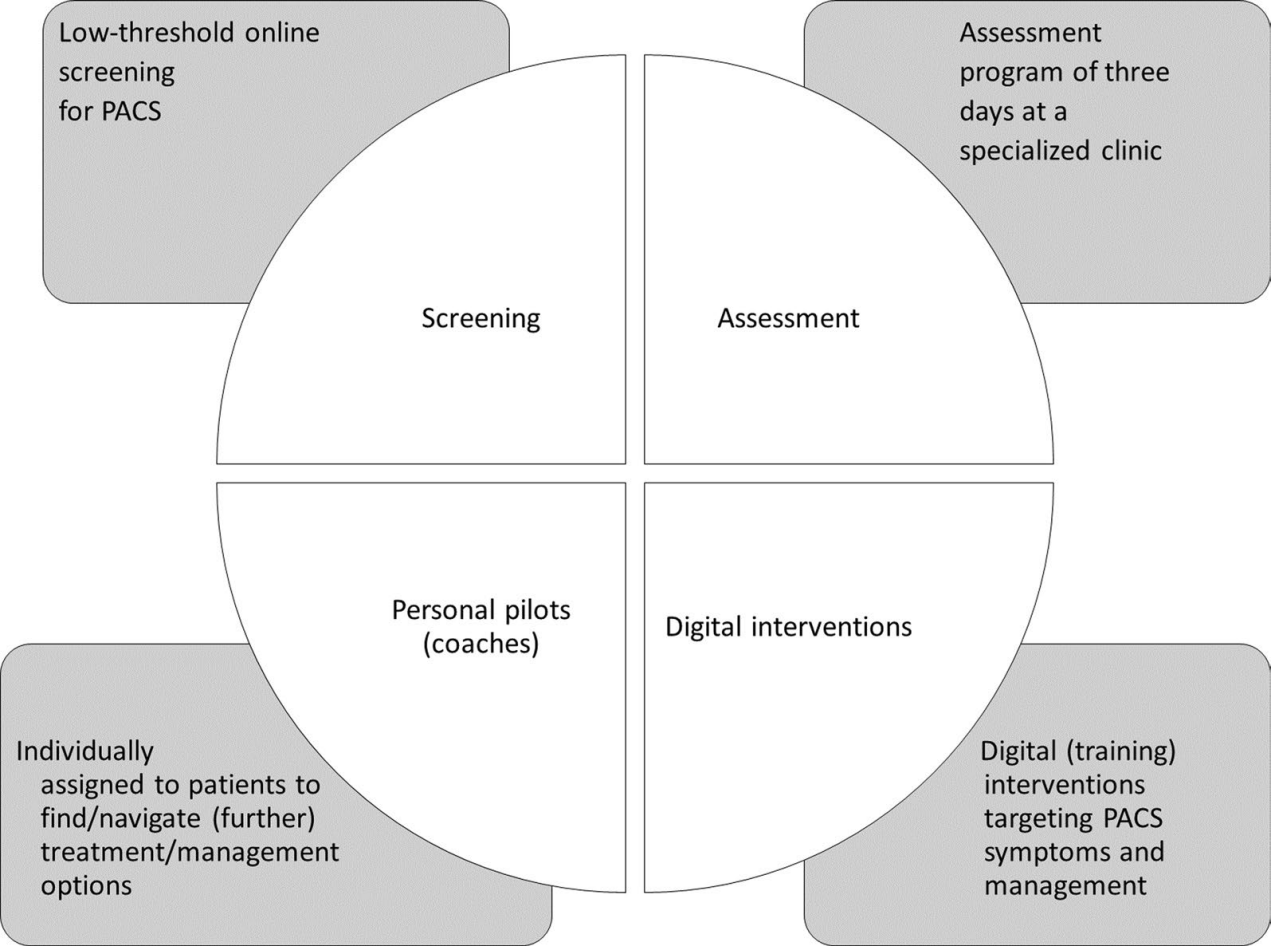
ASAP core components to enhance patient-centered care of post-acute COVID-19 syndromes

In the current project, a comprehensive diagnostic and intervention program is developed including four main categories, outlined in the following figure (Fig. 4).

#### 1. Low-threshold online screening tool

To establish a quick and easy-to-use screening tool for patients who assume that their symptoms are due to PACS, a low-threshold online screening instrument was developed and will be evaluated during the duration of the project (see Additional file 1﻿: Appendix). The screening is of subjective nature. It consists of 14 questions that assess symptoms related to PACS. In addition, the screening includes further questions that assess the diagnosis and timeline of acute COVID-19, limitations in daily life and previous treatment measures to reflect the inclusion and exclusion criteria. Furthermore, demographic questions are asked about age, sex, and place of residence.

If the responses indicate the likelihood of PACS, and that the participants meet the inclusion criteria (Table 1), participants are asked to provide their contact details so that they can be contacted by the personal pilot and take part in the study. In this case, patients are randomly assigned to either the intervention group (IG) or the active control group (ACG). If a randomized approach is not possible (e.g., if it is not possible for a patient to take part in the three-day assessment program), they will not be randomly assigned but marked for the statistical analyses. If there is no indication of a PACS in the screening tool, participants will be directly informed of this, and advised to contact their GP for further clarification and support. In the scope of the project, the online screening will be validated with the results of the assessment program to ensure that the screening can reliably identify patients suffering from PACS and inform future interventions. In detail, the results from the assessment will serve as a gold standard for the validation of the online screening. That is, after the assessment, practitioners will be asked to decide to what extent patients are indeed affected by PACS. This decision will be used to be able to determine the sensitivity for the online screening. In case of an acceptable match between the decision of the practitioners and the online screening, the online screening can be used as a starting point for appropriate treatment of patients and Hypothesis 1 that a short screening is able to detect the presence of PACS will be validated.

#### 2. Comprehensive assessment program (intervention group only)

After the randomization and an initial interview with the personal pilot, patients in the IG are assigned to a 3-day inpatient assessment. During the assessment, the functional capacity will be examined by different disciplines according to the bio-psycho-social model of the International Classification of Functioning (ICF) (DIMDI, 2005) and the need for treatment will be identified depending on the debilitating symptoms.

The 3-day inpatient assessment takes place in a specialized clinic (neurological-orthopedic stationary rehabilitation center). This clinic has the necessary (specialist) competence in the fields of neurology and orthopedics including physiotherapy, internal medicine, and psychology. The assessment aims to check in more detail and on basis of objective tests for the presence of PACS. Furthermore, the goal is to determine an individual interdisciplinary and cross-sectoral treatment plan, which can be translated into practice. This plan is then guided by the personal pilot in terms of empowering the patients to know about their symptoms, the recommended treatments, and what they can do themselves also by means of the digital training.

Concretely, the assessment is performed by means of physiological and psychological tests (see Table 2). These tests are based on the recommendations of the S1-guidelines (e.g., [17]) and consist also of a detailed medical history and review. From this, further individual assessments are derived adaptively. They are carried out by different therapists from the professional groups of pneumology, cardiology, internist, psychology, neurology, physiotherapy, occupational therapy, and nursing.

**Table 2:** Diagnostic tools within the assessment

Domain	Parameter/ tests
Physical conditions and vital signs	• Height • Weight • Body fat • Pulse • Electrocardiography at rest • Oxygen saturation • Body temperature • Blood pressure • Breathing frequency
Lung	• Spirometry
Brain	• EEG • Initial neurological examination
Nerves	• Nerve conduction velocity • Somatosensory evoked potentials (SEP)
Stress test	• Schellong orthostasis test • Ergometry
Taste/smell	• Taste/smell tests (SS-16)
Psychological	• Attention test battery • Alertness test • California Verbal Learning test • Five Point test (divergent thinking) • Visual scanning test • Working memory test
Ability-to-act investigation	• Occupational therapy
Internal diagnostics	• Echography • Carotis-duplex sonography

The results of the assessment for each professional group will be handed to a clinician, who is then able to make a holistic assessment of whether PACS is present or not. These assessments are also summarized to form a holistic treatment plan, which synopsizes the findings and lists the indicated medical and therapeutic measures. These recommendations will be made visible for the personal pilot, and subsequently made available to the attending outpatient doctor.

The prescription of necessary treatments and aids, as well as the recommendation of digital interventions already take place within the framework of discharge management. If the need for a rehabilitation measure is determined, this will be communicated to the patient directly in a way to enable him/her applying for rehabilitation treatment and finding the most appropriate treatment within Germany (also with the help of the personal pilot).

Using such an approach allows us to test Hypothesis 2, that a comprehensive assessment can determine rehabilitation or remedial needs and the leading rehabilitation indication. To assess the applicability of this hypothesis, we will consider a comparison of test–retest reliabilities of the degree of experienced symptoms in the IG compared with the ACG and the CompG. Furthermore, the assessment allows us also to test Hypothesis 5 that an interdisciplinary treatment pathway empowers the patient. To assess the applicability of this hypothesis we will check whether IG, ACG and CompG differ in terms of the development of symptoms, increased activities of daily lives, RTW and increased social participation. To do so, we will consider MANOVA and repeated measurement ANOVA analyses.

#### 3. Digital therapy offers

During the entire care process and after the onboarding performed by the personal pilots, digital intervention in terms of physical training courses and psychological interventions are provided to the patients. Patients can take part individually, regardless of location and time (so-called asynchronous offers [30]). The digital therapy services will support the treatment and bridge any waiting times for the patients in the IG to get the assessment and other therapies, as well as to support any indicated rehabilitation measures (cf. [33]). Patients in the IG will receive digital interventions on the basis of their physical resilience and patients in the ACG will receive one of three digital interventions based on their leading symptoms (fatigue, cardio-/respiratory, neuro-psychiatric).

In addition to the content of the digital tool, an interactive chatbot will be developed and tested during the duration of the ASAP project. The chatbot will focus more on short interventions targeting the management of symptoms in daily life, including self-discrepancies, pacing, and coping.

The patients will receive information and training to initiate the treatment of the present symptoms and to strengthen their self-management as well as to contribute to psychological stabilization. Contents include training in relaxation/breathing exercises, autogenic training, mindfulness/relaxation exercises, meditation, muscle relaxation as well as psychoeducation, strength training, physical endurance and coordination training, sensory training (including smell and taste), functional training as well as training on how to deal with the disease in an occupational context (cf. [34]).

Using this method and determining differences in the IG (patients who receive individualized digital offers on basis of their physical resilience), ACG (patients who received individualized  offers based on the most debilitating symptoms) and CompG (patients who only received the chatbot on a voluntary basis) in the questions about how effective they found the digital interventions in the treatment of PACS allows us to test Hypothesis 3. Within this hypothesis, it is assumed that the more individualizable digital interventions are, the better they work. To assess this, we will check whether IG, ACG and CompG differ in terms of the development of symptoms, increased activities of daily lives, RTW and increase social participation with individualization as another factor. To do so, we will consider MANOVA and repeated measurement ANOVA analyses considering individualization accordingly.

#### 4. Personal pilot

The personal pilots are the first point of contact for all patients who are admitted to the project. Pilots have the qualifications of trained psychologists, social workers/educators, health scientists or health care professionals. They ensure the connection between the project management team providing the outcome from the screening, the specialized clinic conducting the assessment and the patients—both in an anonymized way. A personal pilot is assigned to each patient who was screened positively and who takes part in the study.

The personal pilots contact the patient by phone or email within 4 days after the participant has finished the screening tool. They provide information about the study process. The personal pilot arranges the inpatient assessment in the IG by making the referral to the assessment clinic. In addition, the personal pilot recommends and organizes participation in digital services.

The personal pilot is available throughout the entire process of care, from screening to implementation of the treatment plan. Thus, the pilot supports the patients throughout the entire medical care as a contact point for both the patients and the medical service providers involved. At regular intervals, they contact the study participants in order to reflect on the progress, make qualitative notes of their observations with the patients (e.g., whether there was anything special or unusual to discuss, or what else patients need or want beyond the [digital] interventions that are already provided in the study) and try to motivate the patients also to increase the study commitment of the patients. Their final aim is to empower patients in their coping with PACS and enable them to find and make use of the best treatment. In the final discussion at the end of the study participation, the personal pilot gives recommendations for further care. The personal pilots are trained in motivational interviewing [35, 36].

The personal pilot supports the referral to any necessary further diagnostics and therapy in the IG and ACG (e.g., therapies, rehabilitation, aftercare) and helps the patient to identify suitable leisure activities, such as individually appropriate physical exercises, gyms and recreation/fitness centers close to the patient’s home. The pilots help to arrange the appropriate contacts and access to suitable self-help groups. Moreover, the pilot helps to find a general practitioner if none is available yet. The personal pilot serves as an empowering coordinator throughout the entire care process, mediating information to the various service providers in the inpatient and outpatient sectors and the patients not admitted to the study by means of dissemination strategies.

With the use of personal pilots, we will assess the applicability of Hypothesis 4 that a personal pilot can ensure the sustainable management of interdisciplinary treatment. To test this hypothesis, we will use a qualitative approach and ask the personal pilots to what extent patients have engaged in sustainable treatment and what factors in the interaction between the pilots and the patients were especially helpful to foster a sustainable treatment. Accordingly, answers will be analyzed by means of qualitative content analysis [37–39].

### Evaluation concept

A longitudinal randomized control trial design is used in this project with a three-group pretest–posttest procedure. Whereas the CompG will be recruited with different links via online invitations (e.g., via social media campaigns), the IG and the ACG are recruited with another separate link. After agreeing to take part in the study, participants will be randomized by throwing a dice. Patients for whom a 1 or 2 was rolled are placed in the IG and patients for whom a 3–6 was rolled are placed in the ACG. If a randomization cannot be realized (e.g., because they cannot take part in the stationary assessment due to organizational limitations), patients will be allocated to the ACG but will be marked for statistical analyses. Methodically, a propensity score matching will be used between the IG and the CompG to ensure the comparability of these groups (i.e., participants will statistically be matched by various person-related criteria such as age and sex). All groups are repeatedly interviewed with similar instruments in each group (see Table 1). Time points of measurement are the screening (T1), prior to the assessment in the IG and a comparable time point in the ACG (T2), after the assessment in the IG and at a comparable time point in the ACG (T3). Then approximately 6 weeks after T3 and after symptom-specific digital interventions, a further measurement will take place (T4). Another 6 weeks later a follow-up measurement will take place (T5). A last measurement time point (T6) will be conducted 6 months later with some selected participants. All time points of measurement are included in the study flow chart (Fig. 3).

As the project aims to evaluate the four components (online screening, personal pilots, assessment, digital interventions, see Fig. 4) and the five hypotheses that were described above, a structured evaluation plan is set up. The study aims to alleviate or resolve PACS, and thus to generally restore physical and mental functioning. In addition, the aim is to resume performance in terms of activity and social participation. Within the framework of a successful recovery process as described in the CCAM (see Fig. 2), the active cooperation of the patient affected is obligatory. That is, the factors motivation, psychological stability/resilience, and coping strategies will also be considered in the evaluation process. Furthermore, the economic situation and personal contextual factors will also be considered. Different indicators are used to conduct the evaluation process. These include participation/drop-out rates at the different measurement points, acceptance, and satisfaction over the course of participation, implementation fidelity, implementation dose, target group achievement as well as a systematic analysis of the mediating processes.

Focus groups or individual interviews with the clinic staff (at least one person per occupational group) and the participating patients are planned with partially standardized guidelines, which are evaluated with the use of a content analysis. In addition, structured, participatory observations are conducted, and the observations are tracked. The process evaluation is intended to provide information on the implementation fidelity (e.g., which contents were implemented and realized as planned?), and implementation dose (e.g., to what extent were all intended contents covered or implemented? Attendance of participants?).

For the *summative effectiveness evaluation*, the primary endpoint is the symptom reduction regarding PACS, and RTW. Secondary outcomes include social participation, health-promoting behaviors, and activities of daily living (ADL) as well as mental health and life satisfaction/quality of life. Survey and documentation of all endpoints are conducted by means of questionnaires and/or interviews with study participants who have completed interventions over the course of the project as described above. In addition, possible moderators (especially socio-demographic data) and mediators (e.g., motivation, self-efficacy, and coping strategies) are quantitatively reviewed and added to the analyses. An overview of the measures is provided in Table 2 and Additional file 1.

### Statistical analysis

#### Sample size planning

With an alpha error of 0.05 and a power of 68%, a sample of *N* = 132 (*n* = 66 in the IG and *n* = 66 in the ACG) is required to be able to demonstrate an effect of *d* = 0.15. The evaluation will be conducted statistically using a Multivariate Analysis of Variance (repeated-measures MANOVA: within-between interaction; power analysis calculated with G*Power). In addition to classical significance tests (*p*-value of a two-sided test below 0.05), the clinical significance will be calculated with effect sizes.

#### Type of statistical analysis used

To test the aforementioned hypotheses, first, a propensity score matching will be applied between the IG and the CompG. Sensitivity analyses and test–retest reliabilities will be used to test Hypothesis 1 and 2 respectively. To test Hypothesis 3, MANOVA and repeated measurement ANOVAs will be used. To assess Hypothesis 4, no statistical analyses but a qualitative approach will be used. For the last hypothesis (Hypothesis 5), repeated measurement ANOVAs and MANOVAs will be used.

## Discussion

The study aims to implement a comprehensive care process for people with post-acute COVID-19 syndrome (PACS) based on theory and evidence. This includes four components: (a) a low-threshold online screening to identify as many individuals as possible with a possible PACS, (b) a physiological and psychological assessment as well as (c) digital interventions as supporting formats within a support system over several weeks and (d) personal pilots supporting the patients to make effective use of the health care system by means of empowerment and guidance. The entire process is aimed at supporting the patient through a personal pilot who acts as the first point of contact for the patient. The personal pilot also coordinates and implements the treatment plan, and after the interventions, supports the patient to find additional and potentially necessary treatment options (i.e., rehabilitation treatment, psychotherapy treatments or other treatment options). In addition, the pilot acts as an executive to also inform all the medical service providers that are included in the screening, assessment, and provision of digital interventions by means of dissemination.

### Dissemination of the study results

After completion of the study project, a large network of outpatient and inpatient providers will have been established who offer the treatment to patients with suspected PACS. If the effects of the personal pilot and the digital training interventions transpire successfully, this provides evidence that such a support system may pay off, especially in times of people in need where no services are available at their convenience.

The aim of the current study is to enable the decision-making processes and the evidence-based treatment of PACS patients in the general population with their diverse and individual complaints in the long term, to discharge them from treatment into their everyday lives, and to reintegrate into society. To do so, a *manual* that includes a description of the contact session between the personal pilot and the patient will be made freely available after the completion of the project to ensure continuous and standardized care according to a well-established and valid process for individuals diagnosed with PACS. To ensure the future care for individuals with PACS, any doctor can introduce patients into the treatment pathway; we believe that what is needed is contact with the personal pilot. Service providers who meet the professional and structural requirements for interdisciplinary and holistic assessment and treatment planning in the sense of the bio-psycho-social model can guide the treatment with a high level of quality.

The comprehensive care process should be able to be transferred to regular care and other illnesses and, after the study is completed, will be presented in a corresponding concept (*white paper*) for diagnostics and care specifically for the target group along with the manual for the personal pilots. Our project, which is intended to close a relevant gap in care, has the potential to not only be transferable regionally, but nationwide and internationally.

The results regarding the hypotheses will be disseminated in scientific publications, which will be communicated to experts and the public by means of press work and social media.

This study will also provide valuable information on the effectiveness, user acceptability, and feasibility of the different components. It addresses the need to investigate new approaches to improve health care provision and support of vulnerable patients, which can relieve health systems from the growing demands caused by challenges with communication around the world and in all areas of medicine and public health.

## Supplementary Information

**Additional file 1:** Appendix low-threshold online screening for PACS.

## Data Availability

The data for this study will not be publicly available due to data protection guidelines. The data will be available on reasonable request from the corresponding author and stored on secured servers of the Jacobs University Bremen gGmbH. Newly developed interventions will be made available on open access servers by the end of the project.

## Abbreviations

ACG: Active control group
ADL: Activities of daily living
ASAP: Assistierter Sofortiger Augmentierter Post-/Long-COVID Plan [Assisted Immediate Augmented Post-/Long-COVID Plan]
CCAM: Compensatory Carry-Over Action Model
CompG: Comparison group
GP: General practitioner
ICF: International Classification of Functioning
IG: Intervention group
NICE: UK National Institute for Health and Care excellence
PACS: Post-acute COVID-19 syndrome
RTW: Return to work
SEP: Somatosensory evoked potentials
SNAP: Smoking, nutrition, alcohol, physical activity
