# Supplementary material for: Screening and assessment for post-acute COVID-19 syndrome (PACS), guidance by personal pilots and support with individual digital trainings within intersectoral care: a study protocol of a randomized controlled trial
Source: BMC Infect Dis. 2022 Aug 15;22:693. doi: 10.1186/s12879-022-07584-z (PMC9377288; doi:10.1186/s12879-022-07584-z)
Supplement: Supplementary file 1 — Additional file 1: Appendix low-threshold online screening for PACS. [file 12879_2022_7584_MOESM1_ESM.docx]

## Appendix

The online screening tool.

All patients fill out the first question. Depending on what they indicate, they will either be directed to block 1 (if they indicate “yes”), to block 2 (if they indicate no, but that they may be infected with COVID 19) or if participants indicate that they were not infected at all, they will be directed to the symptoms block as a control group.

Note the special feature in block 1: If participants do not indicate any question that directly leads to question 4, they will be directed to block 2. Otherwise, they complete block 1 and will be directed to the symptoms block afterwards.

After completing block 2, participants will also be directed to the symptoms block and then all participants will be directed to the general block.

Note: If patients indicate an italic option, they proceed further with the questionnaire but at the end of the questionnaire they receive the feedback that they are not eligible for the study. Italic bold statements with arrows are notes to researchers/health professionals that aim to use this tool.

| ***Start: Question 1***  **Have you been tested positive for the SARS-CoV-2 virus (coronavirus) (since the beginning of the corona pandemic)?** |
| --- |
| Yes (🡪 go to block 1) |
| No, but I am sure that I have had a SARS-CoV-2 infection (🡪 go to block 2) |
| No, but I suspect I may have had a SARS-CoV-2 infection (🡪 go to block 2) |
| *No, and I don't think I had a SARS-CoV-2 infection either (🡪 go directly to the block “symptoms”) (exclusion)* |

| ***Block 1 (the “yes” path)***   1. **How often have you tested positive?** |
| --- |
| 1 time |
| 2 times |
| 3 times |
| More often than 3 times |
|  |
| 1. **Please think back to your SARS-CoV-2 infection(s). Have you received a positive PCR test result at least once?** |
| Yes (🡪 go directly to question 4) |
| No |
|  |
| 1. **Have you performed rapid tests?** |
| No, I have not |
| Yes, exactly one rapid test was positive for me |
| Yes, exactly two rapid tests were positive for me (🡪 go directly to question 4) |
| Yes, more than two rapid tests were positive (🡪 go directly to question 4) |
| ***🡺 Remember: When participants did not indicate an option that leads to question 4, they will be directed to block 2. Otherwise they will be directed to the symptoms block.*** |
| 1. **Taking your symptoms together, could you explain them by something else (e.g., an accident, another illness, or lack of training)?** |
| *Yes, definitely (exclusion)* |
| *Yes, maybe (exclusion)* |
| Yes, but I am not sure |
| Rather not |
| Definitely not |
|  |
| 1. **If you tested positive, when was the last positive test (e.g., a positive PCR test or 2 positive rapid tests)?** |
| *Less than 1 month ago (exclusion)* |
| 1 to 2 months ago |
| 2 to 3 months ago |
| 3 months ago or more |
| I did not test positive. |
| ***🡺 After answering the questions from this block, participants proceed with the symptoms block*** |

| ***Block 2 (the “no” path)***   1. **When did you first have or assume you had SARS-CoV-2 infection?** |
| --- |
| *Less than 1 month ago (exclusion)* |
| 1 month or more but less than 2 months ago |
| 2 months or more but less than 3 months ago |
| 3 months ago or longer |
|  |
| 1. **What do you think is a sign that you were infected with SARS-CoV-2? (You can click on more than one answer).** |
| I have felt sick, like I have the flu or a cold. |
| I had/ have experienced a loss or worsening of my sense of smell and/ or taste. |
| I have become short of breath and/or have a harder time breathing since the infection or shortly thereafter. |
| I have had contact with a person(s) infected with SARS-CoV-2 and have felt sick afterwards. |
| I have noticed various symptoms such as loss of pleasure, and/ or increased fatigue and/ or increased hearing problems. |
|  |
| 1. **Taking your symptoms together, could you explain them by something else (e.g., an accident, another illness, or lack of training)?** |
| *Yes, definitely (exclusion)* |
| *Yes, maybe (exclusion)* |
| Yes, but I am not sure |
| Rather not |
| Definitely not |
| ***🡺 After answering the questions from this block, all participants proceed with the symptoms block*** |

| ***Symptoms block***  **Below is a list of health problems. What about you: Please indicate how problematic these have been or are for you.** |
| --- |
| Answer on scale from 0 = No problem to 4 = Extreme problem |
| 1. sleep disturbances e.g., problems with falling asleep or sleeping through the night as well as waking up early |
| 2. intestinal dysfunction e.g., diarrhea, and constipation |
| 3. joint pain/ muscle pain |
| 4. fever |
| 5. impairment of sense of smell/taste e.g., decreased or altered sense of smell |
| 6. shortness of breath |
| 7. cough |
| 8. hair loss |
| 9. rash |
| 10. fatigue (exhaustion, tiredness...) |
| 11. palpitations (heart racing) |
| 12. limbs falling asleep |
| 13. balance problems and/or disturbances of equilibrium |
| 14. brain fog: memory/ concentration problems and/ or forgetfulness |
| ***🡺 Here, at least 3 symptoms must be rated as greater than 2 otherwise the participants will be excluded from the study*** |

| ***General block (to be filled in by all participants equally)***  **Please think back again to the symptoms you stated before: Do you feel that the symptoms are new, that is, they occurred only because of or after your (possible) SARS-CoV-2 infection? (If you do not think you are infected, please indicate "No, I had the symptoms before too").** |
| --- |
| Yes, I did not have the symptoms before. |
| No, the symptoms are not new. However, they have gotten worse since the infection. |
| No, I had the symptoms before, too. |
| *Yes, I did not have the symptoms before. (exclusion)* |
|  |
| **Do you notice an overall improvement in your general health since your COVID-19 infection - in other words, are you doing better?** |
| *Yes, I feel much better than I did a few days or weeks ago and I am confident that I will recover completely. (exclusion)* |
| Yes, I feel much better than I did a few days or weeks ago. However, I still feel bad and I am not sure that I will get completely well again. |
| No, the symptoms have been going on for some time. |
| No, since my SARS-CoV-2 infection, my health has changed, but I have an overall feeling that it is not improving. |
|  |
| **To summarize again: How much are you currently affected in your daily life by the consequences of your COVID-19 symptoms?** |
| *I have no limitations in my daily life and no symptoms, pain, or anxiety related to the infection. (exclusion)* |
| I have negligible limitations in my daily life as I can perform all usual duties/activities, although I still have persistent symptoms, pain, or anxiety. |
| I suffer limitations in my daily life as I occasionally have to avoid or reduce usual duties/activities or spread them over time due to symptoms, pain, or anxiety. However, I am able to perform all activities without any assistance. |
| I suffer from severe limitations in my daily life: I am unable to care for myself without assistance and depend on the care and/or help of another person due to symptoms, pain, or anxiety. |
|  |
| **What treatment interventions have you received for your post/Long COVID symptomatology? (If you have not received any so far, do not tick any of the answers). You may check more than one answer.** |
| *Inpatient rehabilitation following hospitalization (AR, AHB). (exclusion)* |
| *Full-day out-patient rehabilitation (exclusion)* |
| *Outpatient therapy or remedies (e.g., physiotherapy) (exclusion)* |
| Provision of medical aids |
|  |
| **What is your biological sex?** |
| Male |
| Female |
| Others |
|  |
| **How old are you?** |
| _____________ |
| ***🡺 Note: Patients who are younger than 18 years and older than 60 years old will be excluded from the study*** |
|  |
| **Is your primary residence in Bavaria?** |
| Yes |
| *No* |
|  |
| **Which care degree do you have? *(Note: In Germany a care degree is given to individuals with a certain degree of limitations due to chronical health conditions. Whereas a degree of 1 indicates a minor impairment of independence, a degree of 5 indicates severe limitations of independence. This group of individuals need special care).*** |
| I do not have a care degree |
| degree 1 |
| *degree 2 (exclusion)* |
| *degree 3 (exclusion)* |
| *degree 4 (exclusion)* |
| *degree 5 (exclusion)* |
